# The prognostic and clinicopathological value of HALP score in non-small cell lung cancer

**DOI:** 10.3389/fimmu.2025.1576326

**Published:** 2025-06-26

**Authors:** Qin Li, Mengqi Chen, Huaqin Zhao, Jiawei Zeng

**Affiliations:** ^1^ Department of Clinical Laboratory, Mianyang Central Hospital, School of Medicine, University of Electronic Science and Technology of China, Mianyang, Sichuan, China; ^2^ Department of Respiratory and Critical Care Medicine, the Second People’s Hospital of Deyang City, Deyang, Sichuan, China

**Keywords:** HALP score, NSCLC, prognostic, survival, biomarker

## Abstract

**Objective:**

The prognostic role of the hemoglobin, albumin, lymphocyte, and platelet (HALP) score in non-small cell lung cancer (NSCLC) has been widely reported, but the results remain controversial. Therefore, we aim to evaluate the prognostic and clinicopathological value of the HALP score in NSCLC through a pooled analysis.

**Methods:**

We conducted a comprehensive literature search of PubMed, Embase, Web of Science, Cochrane Library, and the ClinicalTrials.gov databases in December 2024 to identify studies evaluating the relationship between the pretreatment HALP score and outcomes in NSCLC patients. Eligible studies included patients treated with surgical resection, chemotherapy, or immunotherapy. The HALP score was calculated using peripheral blood levels of hemoglobin, albumin, lymphocytes, and platelets measured before treatment. Data were extracted and analyzed to determine the association of the HALP score with overall survival (OS), disease/progression/recurrence-free survival (DFS/PFS/RFS), and clinicopathological characteristics. Subgroup and sensitivity analyses were performed to ensure the robustness and reliability of the results.

**Results:**

A total of 10 studies involving 7024 patients were included. The results demonstrated that patients with lower pretreatment HALP score had worse OS (hazard ratio [HR] = 1.73, 95% confidence interval [95% CI]: 1.27-2.34, p < 0.001) and DFS/PFS/RFS (HR = 1.86, 95% CI: 1.30-2.64, p < 0.001). The results remained consistent across subgroup analyses based on study characteristics and sensitivity analyses. Additionally, a lower HALP score was significantly associated with age (odds ratio [OR] = 1.43, 95% CI: 1.15-1.78, p = 0.001) and tumor size (OR = 0.54, 95% CI: 0.38-0.76, p < 0.001).

**Conclusions:**

The HALP score is a valuable prognostic biomarker for predicting survival outcomes in NSCLC patients. Its ability to integrate multiple aspects of systemic inflammation and nutritional status makes it a promising tool for improving risk stratification and guiding treatment decisions. Future studies should continue to validate this finding in prospective, multicentre trials.

## 1 Introduction

Lung cancer is the most common type of cancer worldwide, with an estimated 2.5 million new cases and over 800,000 deaths reported globally in 2022 (1). Non-small cell lung cancer (NSCLC), the most common type of lung cancer, accounts for 85% of all cancer cases (2). Despite the emergence of new therapeutic strategies in recent years, including targeted therapies and immunotherapies for driver mutations, which have led to improved patient survival, the prognosis of the NSCLC remains worrisome (3). Studies have shown that the survival of NSCLC patients is influenced by various factors, including tumor stage, grade, treatment strategy, and the patient’s overall condition (4). Additionally, the heterogeneity in cancer patient prognosis has led to confusion among clinicians. Therefore, identifying effective biomarkers or scoring systems to predict prognosis is crucial for improving patient prognosis assessment and developing personalized treatment plans.

Research has demonstrated that inflammation plays an indispensable role in cancer development, tumor angiogenesis, and metastasis (5). In recent years, several inflammatory biomarkers, such as the platelet-to-lymphocyte ratio (PLR), neutrophil-to-lymphocyte ratio (NLR), and lymphocyte-to-monocyte ratio (MLR), have been demonstrated to be associated with cancer prognosis (5, 6). However, they each reflect only a single inflammatory or immune parameter and are susceptible to non-tumor factors, suggesting that the predictive power of prognosis remains limited (7). The hemoglobin, albumin, lymphocyte, and platelet (HALP) score, as a novel prognostic evaluation tool, has gradually attracted the attention of researchers. Currently, the HALP score has indicated significant potential in prognostic evaluation across various cancers. Chen et al. (8) found that the HALP score could independently predict survival in gastric cancer patients and was closely related to overall prognosis. Preoperative HALP score was also considered an independent prognostic factor for cancer-specific survival in renal cell carcinoma and esophageal cancer (9, 10). These studies have illustrated that the HALP score reflects the patient’s systemic nutritional status, inflammatory response, and immune function and overcomes the limitations of single markers that focus primarily on inflammation, thus providing valuable information for the prognostic assessment of cancer patients.

Despite the prognostic efficacy of the HALP score in NSCLC has been reported, current findings are somewhat controversial, and most studies have small sample sizes, limiting the generalizability of their conclusions (11–13). Therefore, this study aims to evaluate the prognostic role of the HALP score in patients with NSCLC and to explore its correlation with patients’ clinicopathological characteristics through a meta-analysis. This will help provide new reference points for clinical practice and further advance the application research of the HALP score in cancer prognosis assessment.

## 2 Methods

### 2.1 Search strategy

The meta-analysis and systematic review followed the Preferred Reporting Items for Systematic Reviews and Meta-Analyses (PRISMA) and the Assessing the methodological quality of systematic reviews 2 (AMSTAR 2) Guidelines (14, 15). The analysis protocol was prospectively registered in PROSPERO (CRD42024565673). Comprehensive searches were performed across multiple databases including PubMed, Embase, Web of Science, Cochrane Library, and the ClinicalTrials.gov to identify relevant studies published up to December 2024. Search strategies combined both controlled vocabulary and free-text terms, with key terms including “hemoglobin,” “albumin”, “lymphocyte”, “platelet”, “HALP”, “lung neoplasms”, “lung cancer”, “lung carcinoma”, “lung adenocarcinoma”, “non-small cell lung cancer”, and “NSCLC”. We also manually searched the reference lists of relevant reviews and meta-analyses to identify potential literature. Any disagreements were resolved through discussion, with final arbitration by a third author if necessary.

### 2.2 Selection criteria

The two authors conducted literature screening based on the following inclusion criteria: (1) study types included prospective or retrospective cohort studies, (2) study subjects were patients diagnosed with NSCLC, (3) the intervention involved assessing the pretreatment HALP score of patients and dividing them into high and low groups, (4) outcome measures included overall survival (OS), progression/disease/recurrence-free survival (PFS/DFS/RFS), and/or clinicopathological characteristics. Exclusion criteria comprised reviews, case reports, conference abstracts, and commentary articles, as well as duplicate publications, studies not involving NSCLC patients, and studies lacking sufficient data for extraction and analysis.

### 2.3 Data extraction and quality assessment

We extracted data according to predefined criteria, including the following details: study basic information (authors, publication year, country), patient characteristics (gender, sample size, age, smoking history, median follow-up time), intervention measures (therapeutic measures, HALP cut-off, and the method of determining HALP cut-off), survival outcomes (OS, PFS, DFS, and/or RFS), and statistical analysis (hazard Ratio [HR] with its 95% confidence interval [CI]). In this study, we utilized the Newcastle-Ottawa Scale (NOS) to assess the quality of included studies (16), which includes selection of study groups, comparability between groups, and assessment of outcomes. The NOS score ranges from 0 to 9, with studies scoring 7 or higher considered high quality. Two independent researchers conduct this process, and any discrepancies are resolved through discussion or adjudicated by a third researcher.

### 2.4 Statistical analyses

The data analysis was conducted using RevMan 5.4 and Stata 16.0 software. We assessed heterogeneity among studies using the I² statistic and Q test. Heterogeneity was considered significant if I² > 50% or P < 0.1, and a random-effects model was used for meta-analysis; otherwise, a fixed-effects model was employed. HR and their 95% CI served as the effect size metric in this study. To explore potential sources of heterogeneity, we conducted subgroup analyses based on patient characteristics, treatment modalities, and HALP cut-off values. In addition, meta-regression analyses were performed to assess whether covariates contributed to heterogeneity. Sensitivity analysis was carried out by sequentially omitting individual studies to evaluate the robustness and stability of the overall results. Publication bias was assessed using both Egger’s test and Begg’s test, with funnel plots used for visual inspection. In cases where significant publication bias was detected, the trim-and-fill method was applied to estimate the adjusted effect size. All statistical tests were two-sided, and a P-value < 0.05 was considered statistically significant.

## 3 Results

### 3.1 Study selection and characteristics

**Figure 1:**
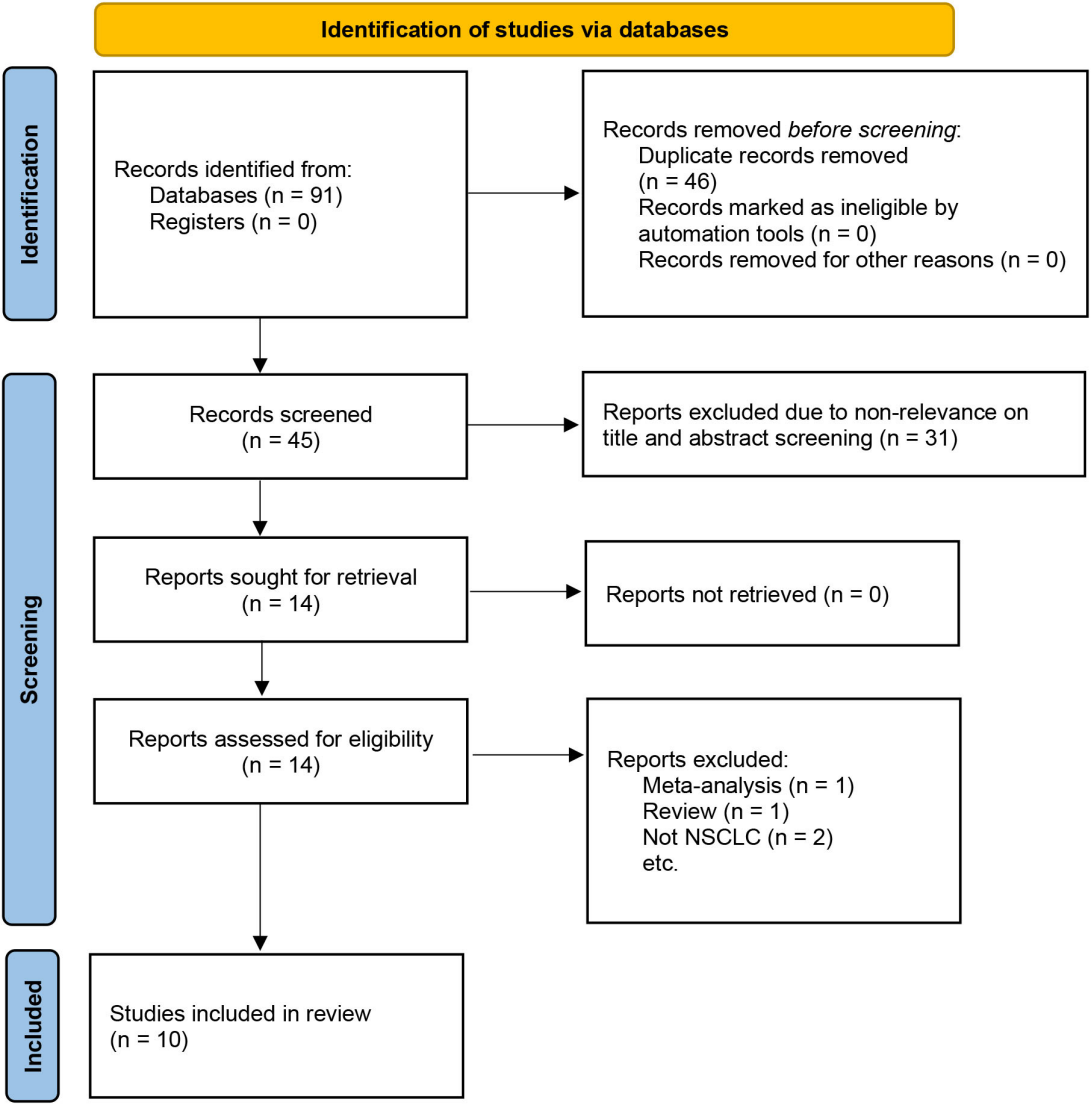
Flow diagram of literature retrieval strategy.

The initial search identified 421 articles, and after removing duplicates, 345 articles remained. By reading the titles and abstracts, 319 articles were excluded. After reading the full texts of 26 articles, 15 were excluded. Finally, 10 articles with 7024 patients were included in the meta-analysis (**Figure 1**) (11–13, 17–23). Among these studies, 5 involved therapeutic resection (11–13, 20, 21), 4 involved adjuvant chemotherapy (17, 18, 22, 23), and one used immunotherapy (19). The included studies were published between 2021 and 2024. Six studies were from China (11, 12, 18, 19, 21, 23), two from Turkey (17, 22), and the remaining two were from Italy and the United Kingdom (13, 20). The sample sizes of the included studies ranged from 52 to 5,029 cases, with HALP cut-off values ranging from 24.3 to 48.2. The median follow-up period of the included studies ranged from 25.3 to 64 months, but one study did not report the follow-up times (11). Additionally, nine studies reported the association between HALP and OS (11–13, 17–20, 22, 23), two studies examined the relationship between HALP and DFS (12, 18), two studies reported the association between HALP and PFS (19, 23), and one study investigated the relationship between HALP and RFS (21). The NOS scores of nine studies were ≥7, indicating that the overall quality of the included studies was high (**Supplementary Table S1**). The main characteristics and quality assessment results of the included studies are summarized in **Table 1**.

**Table 1:** Baseline characteristics of included studies.

Study	Region	Cancer type	Stage	Treatment	Sample size (N)	Mean age (years)	Gender (M/F)	Smoking (ever/never)	Cut-off	Outcomes	Follow-up (months)	NOS score
Zhai 2021 (11)	China	NSCLC	IA-IV	Surgery	238	62.3	150/88	87/151	48	OS	NA	7
Güç 2022 (17)	Turkey	NSCLC	NA	Chemotherapy	401	63.47	317/84	310/91	23.24	OS	Median 18	8
Wei 2022 (18)	China	NSCLC	I-IV	Chemotherapy	362	NA	217/145	141/221	48.2	OS, DFS	Median 64	9
Fang 2023 (19)	China	NSCLC	IIIB-IV	Immunotherapy	223	60.4	189/34	NA	39.33	OS, PFS	Median 20.4	6
Mazzella 2023 (20)	Italy	NSCLC	I-III	Surgery	257	NA	149/108	NA	32.2	OS	Median 40	8
Zhao 2023 (21)	China	NSCLC	IA-IIIA	Surgery	219	NA	NA	87/132	29.31	RFS	Median 24	7
Zhang 2023 (11)	China	NSCLC	IA-IIIB	Surgery	52	43-79^a^	35/17	25/27	24.3	OS, DFS	Median 42/36^C^	8
Cavdar 2024 (22)	Turkey	NSCLC	NA	Chemotherapy	278	63^b^	260/18	157/121	26	OS	Median 15.3	8
Gao 2024 (23)	China	NSCLC	IIIB-IV	Chemotherapy	203	59.6	140/63	92/111	28.02	OS, PFS	Median 16	9
Taylor 2024 (13)	UK	NSCLC	I-III	Surgery	5029	68.6	2444/2585	4144/885	36.87	OS	Median 33	8

NSCLC, non-small cell lung cancer; OS, overall survival; PFS, progression-free survival; DFS, disease-free survival; NA, not available;

a Reported as rang;

b Reported as median.^C^40 for OS and 36 for DFS.

### 3.2 HALP and clinicopathological characteristics

**Figure 2:**
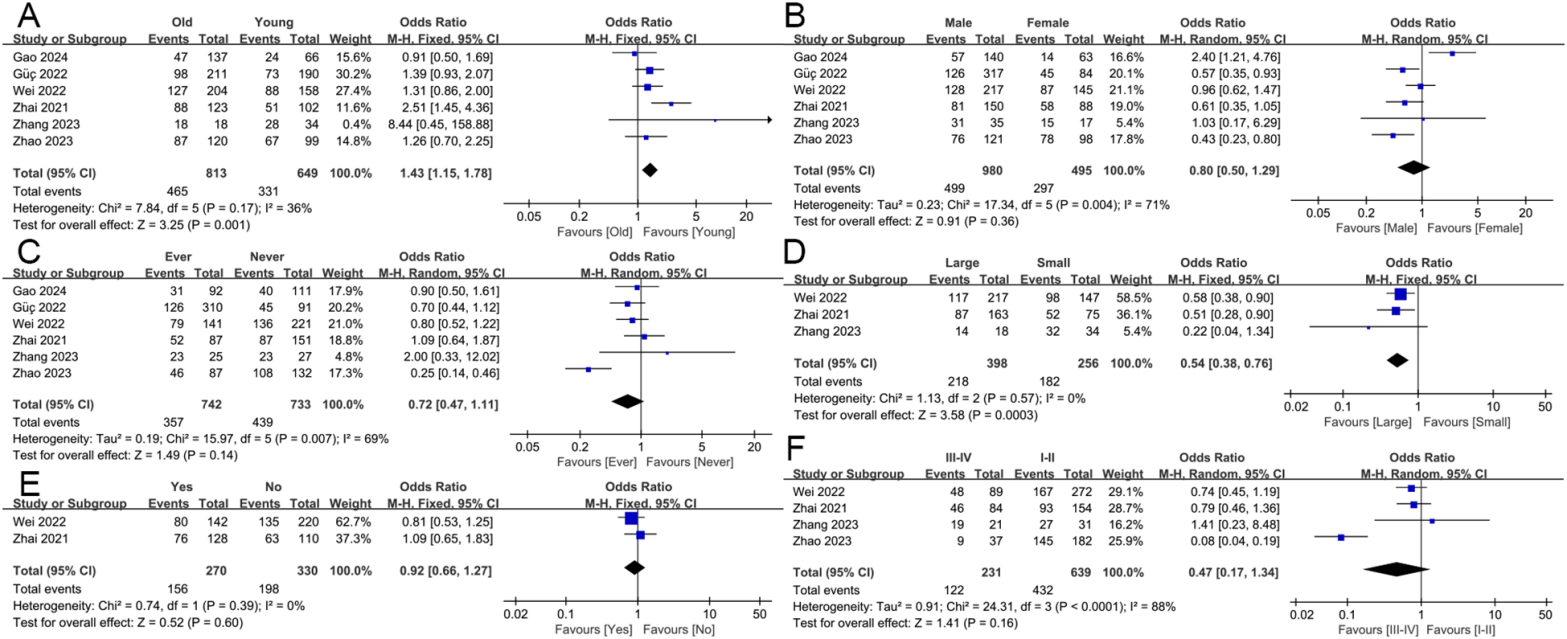
The association of the HALP score with clinicopathological characteristics. including age **(A)**, gender **(B)**, smoking history **(C)**, tumor size **(D)**, lymph node metastasis **(E)**, and overall staging **(F)**.

In this study, we explored the correlation between HALP score and various clinicopathological characteristics, including age, gender, smoking history, tumor size, lymph node metastasis, and overall staging (**Figure 2**). The results indicated that HALP score was significantly associated with age (old vs young: OR = 1.43, 95% CI 1.15-1.78, p = 0.001) and tumor size (large vs small: OR = 0.54, 95% CI 0.38-0.76, p < 0.001), while no significant associations were found with gender (male vs female: OR = 0.80, 95% CI 0.50-1.29, p = 0.36), smoking history (ever vs never: OR = 0.72, 95% CI 0.47-1.11, p = 0.14), lymph node metastasis (yes vs no: OR = 0.92, 95% CI 0.66-1.27, p = 0.60), or overall stage (III-IV vs I-II: OR = 0.47, 95% CI 0.17-1.34, p = 0.16).

### 3.3 HALP and OS

**Figure 3:**
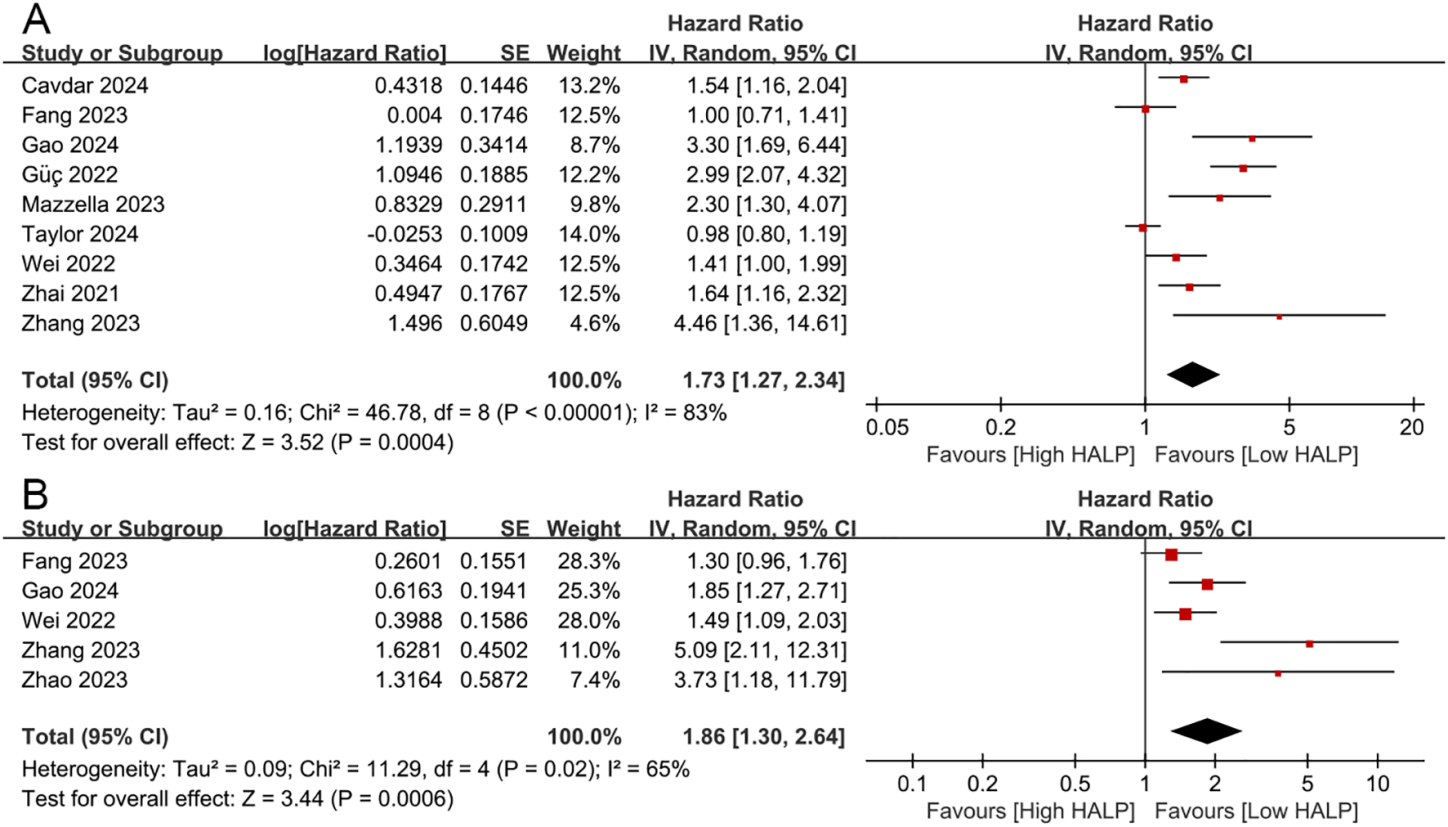
The prognostic impact of the HALP score on overall survival **(A)** and disease/progression/recurrence-free survival **(B)**.

A total of eight studies explored the correlation between HALP score and OS in patients with NSCLC (11–13, 17–20, 22, 23). The pooled analysis revealed that a lower pre-treatment HALP score was significantly associated with poorer OS (HR = 1.73, 95% CI 1.27-2.34, p < 0.001, **Figure 3A**), with substantial heterogeneity observed among the included studies (I^2^ = 83%, p < 0.001). To further investigate potential sources of heterogeneity, subgroup analyses were conducted based on study region, therapeutic strategy, sample size, HALP cut-off value, and follow-up duration (**Table 2**). These subgroup analyses largely supported the overall findings. However, no significant association between the HALP score and OS was observed in the subgroup of studies conducted in other regions (p = 0.40). With respect to cut-off value determination methods, only studies using ROC curve-derived thresholds showed a significant association between low HALP score and poor OS (p < 0.01), while those using X-tile (p = 0.16) or the median value (p = 0.98) did not yield statistically significant results. Regarding therapeutic strategy, in the subgroup of patients receiving chemotherapy, the HALP score was significantly associated with OS (p < 0.01), while in the subgroup of patients undergoing therapeutic resection, the correlation between HALP score and OS was not statistically significant (p = 0.05).

**Table 2:** Results of the primary analysis and subgroup analyses for OS.

Subgroup	No. of studies	HR	95% CI	*P*	Heterogeneity		Meta-regression (*P*)
					I^2^ (%)	*P*	
**Overall**	9	1.73	1.27-2.34	< 0.01	83	< 0.01	
Region							0.759
China Turkey Others	5 2 2	1.70 2.12 1.43	1.15-2.51 1.11-4.06 0.62-3.31	< 0.01 0.02 0.40	72 87 87	< 0.01 < 0.01 < 0.01	
Therapeutic strategy							0.571
Therapeutic resection Chemotherapy Immunotherapy	4 4 1	1.70 2.05 1.00	1.01-2.85 1.37-3.07 0.71-1.41	0.05 < 0.01 0.98	82 77 -	< 0.01 < 0.01 -	
Sample size							0.165
> 238 ≤ 238	5 4	1.66 1.91	1.11-2.50 1.08-3.35	0.01 0.03	88 79	< 0.01 < 0.01	
Cut-off							0.113
> 30.75 ≤ 30.75	6 3	1.48 2.40	1.08-2.04 1.32-4.34	0.02 < 0.01	78 79	< 0.01 < 0.01	
Cut-off determination							0.354
ROC curve X-tile Median	6 2 1	1.87 2.17 1.00	1.25-2.79 0.73-6.46 0.71-1.41	< 0.01 0.16 0.98	87 70 -	< 0.01 0.07 -	
Follow-up period							0.739
> 36 ≤ 36 Not available	3 5 1	2.01 1.64 1.73	1.17-3.46 1.04-2.58 1.27-2.34	0.01 0.03 0.005	58 90 -	0.09 < 0.01 -	

To further evaluate whether these study-level characteristics contributed to heterogeneity, meta-regression analysis was performed. The results indicated that none of the examined variables, including the method used for HALP cut-off determination, significantly explained the heterogeneity (all p > 0.05). This suggests that while subgroup trends exist, the differences observed across subgroups may not be sufficient to account for the overall heterogeneity in a statistically meaningful way.

### 3.4 HALP and DFS/PFS/RFS

Four studies investigated the correlation between HALP score and DFS/PFS/RFS in patients with NSCLC (12, 18, 19, 21, 23). The results revealed that decreased HALP score was significantly associated with worse DFS/PFS/RFS (HR = 1.86, 95% CI 1.30-2.64, p < 0.001, **Figure 3B**), with higher heterogeneity among the studies (I² = 65%, p = 0.02).

### 3.5 Sensitivity analyses

**Figure 4:**
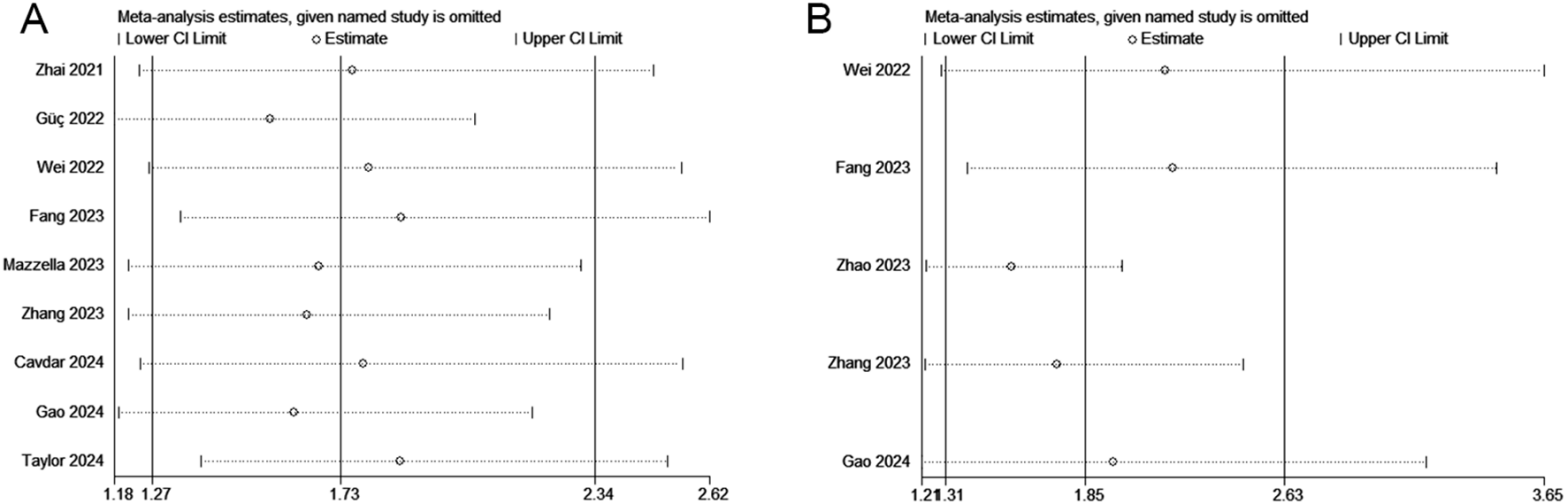
Sensitivity analyses regarding overall survival **(A)** and disease/progression/recurrence-free survival **(B)**.

We performed a sensitivity analysis to assess the reliability of the combined results for OS and DFS/PFS/RFS. The overall HR estimates for both outcomes did not significantly change after removing each study sequentially from the analysis (**Figure 4**), supporting the conclusion that the meta-analysis results are relatively stable and reliable.

### 3.6 Publication bias

**Figure 5:**
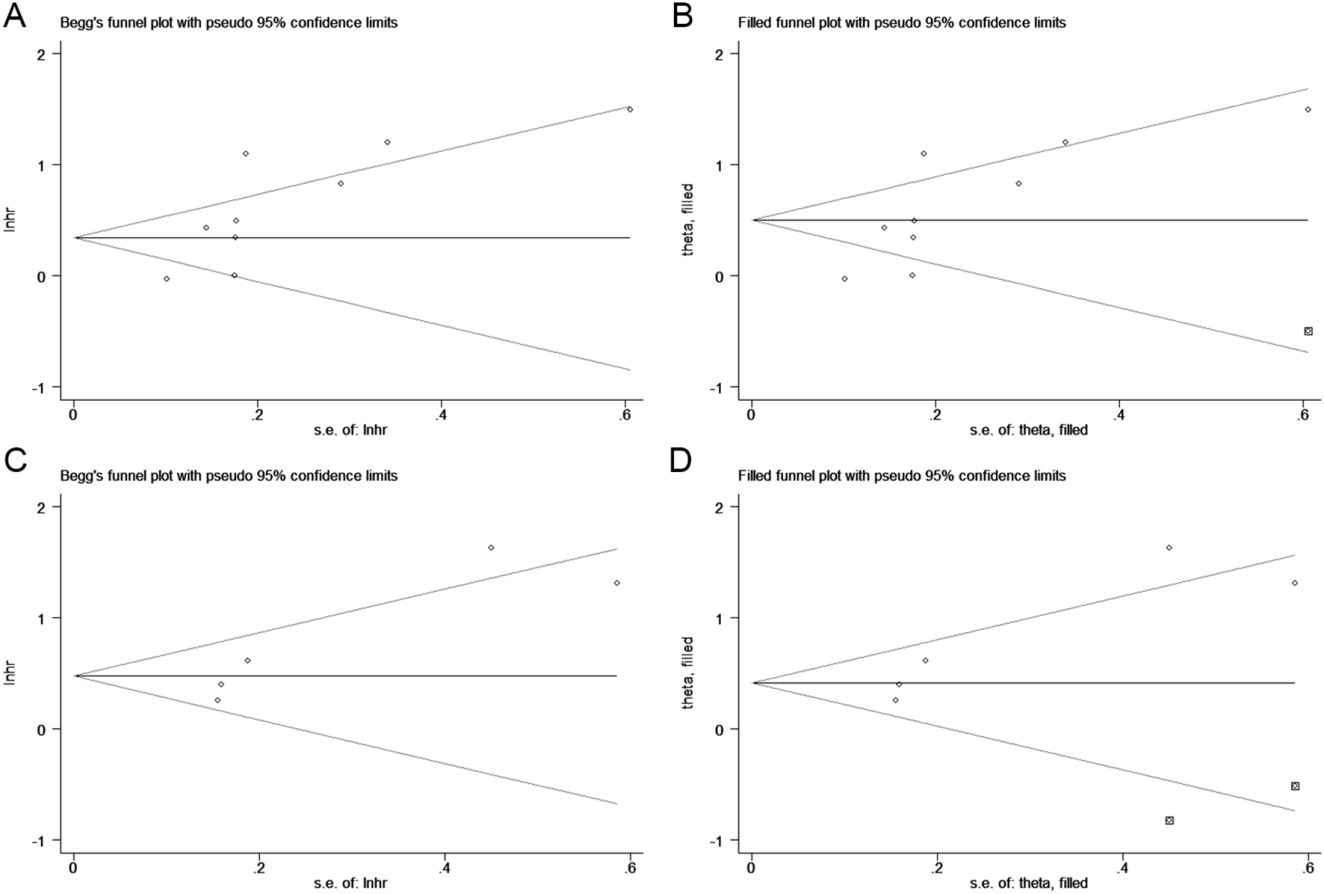
Funnel plots for detecting publication bias in terms of overall survival **(A, B)** and disease/progression/recurrence-free survival **(C, D)**.

We used Begg’s funnel plot and Egger’s test to evaluate publication bias among the included studies. The results indicated asymmetry in the funnel plot, and Egger’s test also showed statistical significance (OS: p = 0.023, DFS/PFS/RFS: p = 0.037, **Figure 5**), suggesting the potential presence of publication bias in our findings. Consequently, we performed the trim-and-fill analysis to adjust for potential publication bias. The results further demonstrated that our results remained robust (OS: HR = 1.651, 95% CI 1.224-2.227, p = 0.001; DFS/PFS/RFS: HR = 1.514, 95% CI 1.017-2.254, p = 0.041).

## 4 Discussion

Despite continuous advancements in diagnostic technologies and therapeutic methods, the incidence and mortality rates of lung cancer remain elevated compared to most other cancers (1, 24). Therefore, there is an urgent need for new biomarkers to predict the prognosis and guide the treatment strategies for lung cancer. As the most common type of lung cancer, the prognosis of NSCLC has been a primary focus for researchers. The HALP score is a novel, simple, and practical biomarker system that evaluates the systemic inflammatory status and nutritional condition of patients by combining hemoglobin levels, albumin levels, lymphocyte counts, and platelet counts (8). Several studies have reported on the prognostic value of the HALP score in NSCLC, but the results are still inconsistent (18–20). Here, we conduct a comprehensive literature review and present the first meta-analysis to evaluate the relationship between the HALP score and the clinicopathological characteristics and prognosis of NSCLC patients, aiming to better inform clinical practice.

Through a comprehensive analysis of multiple studies, we found that a lower pretreatment HALP score was significantly associated with inferior OS and DFS/PFS/RFS. The results remained robust and reliable after subgroup and sensitivity analyses. Residence’s attention was drawn to the fact that in the heterogeneity test, we found a high degree of heterogeneity between studies. To explore potential sources of heterogeneity, we conducted subgroup analyses based on population information and study characteristics. The results did not reveal significant sources of heterogeneity, suggesting that region, sample size, cut-off value, and duration of follow-up may not be the main factors influencing the heterogeneity of the overall effect. In addition, sensitivity analyses showed that the combined effect sizes did not change significantly, either through the exclusion of study with large sample sizes (Taylor et al. (13)), or the exclusion of individual studies one by one, implying that the results of the present analysis have excellent robustness and reliability. Furthermore, this study also indicated that patients with lower HALP score tend to be older and had larger tumor size. These findings suggest that the HALP score could be an extremely valuable biomarker for predicting the survival outcomes in patients with NSCLC and has the potential to improve risk stratification.

The findings of this present study are generally consistent with results from various malignant tumors. Preoperative lower HALP score was significantly associated with poorer OS and PFS in patients with breast cancer (25). Xiong et al. (26) demonstrated that the HALP score has good predictive performance for lymph node metastasis and recurrence in endometrial cancer. Recent studies have also confirmed that the HALP score was an independent prognostic factor for unresectable esophageal squamous cell carcinoma (27). Furthermore, a previous meta-analysis investigated the relationship between HALP and solid tumors (28); however, the limited number of studies included on NSCLC may have reduced the reliability of the results. Therefore, more researches were included in this study and relevant clinicopathological characteristics were analyzed, providing a more comprehensive and reliable evidence base for our conclusions.

Inflammation is the body’s response to tissue damage caused by physical injury, infection, exposure to toxins or other types of trauma (29–31). The body’s inflammatory response causes cellular changes and an immune response that leads to repair of damaged tissue and cell proliferation at the site of the damaged tissue (32). However, a sustained inflammatory response disrupts DNA repair mechanisms and cell cycle checkpoints, leading to chromosomal aberrations and thus promoting cancer development (33). In addition, inflammatory mediators in the inflammatory microenvironment assist tumor cells to proliferate, expand and evade immune surveillance (34). Therefore, immune-inflammation is regarded as a key factor in tumorigenesis and progression (35). The HALP score is considered as a potential predictor of cancer prognosis because it combines four indicators, namely haemoglobin, albumin, lymphocytes and platelets, which can comprehensively reflect the systemic inflammatory response and nutritional status of cancer patients. However, the specific mechanism by which HALP score affects cancer prognosis remains unclear.

Hemoglobin is the main component of red blood cells and is responsible for oxygen transport (36). Low hemoglobin levels are commonly associated with anemia, which leads to tissue hypoxia and can drive tumor cells to adapt to the low-oxygen environment through angiogenesis (37). Anemia may also inhibit anti-tumor immune responses possibly by affecting the immune system, which in turn promotes the escape and growth of tumor cells. Additionally, anemia may affect the overall health status and treatment tolerance of cancer patients to some extent (38). A large body of evidence suggests that low hemoglobin levels are associated with a poorer prognosis in patients with NSCLC (39–41). Serum albumin is an important indicator for assessing host inflammation and nutritional status. Low levels of albumin are common in patients with advanced cancer, suggesting that the body is in a state of chronic malnutrition. Malnutrition weakens the body’s immune response and reduces the effectiveness of anti-tumor therapy, which in turn affects the prognosis of patients (42, 43). Additionally, lymphocytes are regarded as an important component of the immune system, and a reduction in their level often indicates a decline in immune function (44). Tumor cells frequently employ immune evasion mechanisms to inhibit lymphocyte function, thereby promoting tumor progression and metastasis (45). Infiltrating lymphocytes have been reported to significantly affect tumor prognosis and response to chemotherapy (46). Notably, platelets have been shown to play a key role in the metastatic ability of cancer. Platelets can accelerate tumor growth and metastasis by releasing growth factors that promote tumor cell proliferation and angiogenesis (47, 48). Platelets also play an important role in inflammatory processes, and tumor-associated chronic inflammatory responses can exacerbate tumor progression by promoting platelet aggregation and activation, which in turn exacerbates tumor progression (49, 50). Elevated platelet levels have also been shown to be associated with poor prognosis in tumors (51, 52). Therefore, a lower HALP score, which reflects lower levels of hemoglobin, albumin, and lymphocytes, and/or higher platelet levels, is significantly associated with poorer survival in patients.

In recent years, significant progress has been made in prognostic modelling of NSCLC, including key factors such as pathological features, molecular analyses and imaging features (53–56). However, these models have certain limitations. For example, gene expression profiling may be affected by systemic disease (57), and imaging models may be subjective due to physician experience and bias (58). In addition, the high cost and radiation risk of advanced imaging techniques such as PET-CT and MRI limit intensive follow-up and may cause psychological distress (59, 60). Therefore, although these models are effective in predicting prognosis, their invasiveness and high cost make them difficult to disseminate. A routine blood test is a simple, quick and common test that is performed when almost all cancer patients are admitted to hospital. It provides important information about metabolism, inflammation and the internal environment (61, 62), and can be used to guide medication in addition to aiding diagnosis (63). Thus, haematological markers provide a valuable reference for physicians and have great clinical value and potential for practical application (58).

Previous studies have reported that a variety of inflammatory markers, such as NLR and PLR, can be used to predict the prognosis of different malignancies (5, 6). However, these markers reflect only one aspect of a patient’s overall health status, whereas treatment outcomes are influenced by a combination of factors, including the patient’s inflammatory response, nutritional status, and immune function (25). This highlights the importance of a comprehensive and multidimensional approach when assessing a patient’s overall health and prognosis. Since its inception, the HALP score has received much attention as a novel biomarker reflecting systemic inflammation and nutritional status, and has been used to predict a wide range of clinical outcomes in a variety of cancers (64). For instance, recent evidence has demonstrated that the HALP score exhibits superior predictive accuracy for OS in breast cancer patients compared to other commonly used inflammatory and nutritional markers (25). Importantly, in the context of NSCLC, Mazzella et al. (20) reported that the HALP score outperformed established inflammatory markers, such as derived NLR (dNLR) and PLR, in survival prediction. Their ROC curve analysis revealed a higher area under the curve (AUC) for HALP, indicating its stronger prognostic value. Together, these findings support the HALP score as a simple yet valuable prognostic tool that can aid clinical decision-making. Specifically, for newly diagnosed NSCLC patients, the HALP score may provide critical prognostic information to help physicians better evaluate patients’ overall health and survival expectations, thereby facilitating more individualized and optimized treatment strategies. Nevertheless, the independent prognostic value and superiority of the HALP score in NSCLC warrant further validation.

In this analysis, most of the included studies were from the same country (China), which may limit the widespread clinical use of the HALP score. In addition, we also observed that in the subgroup with chemotherapy, patients with lower pre-treatment HALP score had poorer OS, whereas no statistically significant association was found in the subgroup undergoing curative resection or in the single immunotherapy-based study. Indeed, patients selected for surgery tend to have earlier-stage disease and may be less influenced by baseline nutritional/inflammatory status, whereas those requiring chemotherapy (often for more advanced or unresectable tumors) may be more vulnerable to systemic factors captured by HALP. This discrepancy suggests that the prognostic utility of HALP could be context-dependent, providing stronger risk discrimination in the chemotherapy setting. Differences in healthcare systems, patient selection, and treatment protocols can have an impact on the applicability of HALP, especially in the context of studies that are predominantly from the same country. Such regional and practice-pattern variations may limit the generalizability of our findings. Consequently, future research should undertake cross-regional and multicentre collaborations to establish universally applicable HALP thresholds, while accounting for local clinical characteristics and predominant treatment modalities.

This meta-analysis has several limitations that should be acknowledged. First, significant heterogeneity was observed across studies, potentially undermining the robustness and generalizability of the pooled estimates. While subgroup and sensitivity analyses were performed, unmeasured confounding due to differences in patient characteristics, tumor stages, and treatment modalities cannot be entirely excluded. Second, detailed treatment protocols, including surgical approaches and postoperative management, were not uniformly reported across studies, which may limit the interpretability of subgroup analyses. Third, inflammatory biomarkers in peripheral blood may be affected by infection, steroid therapy, or other medications that could not be evaluated in this study. Fourth, methodological differences in HALP calculation and the lack of a standardized cut-off threshold may impair comparability and data synthesis. Fifth, information regarding the management of anemia, such as pre-treatment blood transfusions or nutritional interventions, was not consistently reported in the included studies, which may have influenced HALP scores and should be considered in future prospective research. Lastly, some studies had relatively short follow-up durations, which may be insufficient to capture long-term survival outcomes associated with HALP. Future prospective, multicenter studies with standardized protocols, comprehensive data collection, and longer follow-up are warranted to validate and refine the prognostic utility of HALP in NSCLC. In addition, potential interventions aimed at improving HALP components—such as nutritional support, correction of anemia, and anti-inflammatory strategies—should be further investigated to determine whether optimizing the HALP score may translate into improved survival outcomes.

## 5 Conclusions

In summary, our pooled analysis indicates that lower pre-treatment HALP score are associated with poorer survival outcomes in NSCLC patients. These findings highlight the potential of the HALP score as an effective prognostic biomarker, providing crucial prognostic information for NSCLC patients. Future studies should continue to validate these findings in diverse patient populations and in prospective, multicentre trials, and explore the integration of HALP score with other prognostic markers to further refine risk stratification and treatment approaches.

## Data Availability

The original contributions presented in the study are included in the article/**Supplementary Material**. Further inquiries can be directed to the corresponding author.
