# Supplementary material for: The prognostic and clinicopathological value of HALP score in non-small cell lung cancer
Source: Front Immunol. 2025 Jun 26;16:1576326. doi: 10.3389/fimmu.2025.1576326 (PMC12240774; doi:10.3389/fimmu.2025.1576326)
Supplement: Supplementary file 1 [file Table1.docx]

| **Supplementary table 1.** Quality evaluation of the eligible studies with Newcastle–Ottawa scale. | | | | | | | | | |
| --- | --- | --- | --- | --- | --- | --- | --- | --- | --- |
| Assessment criteria | **Selection** | | | | **Comparability** | | **Outcome** | | |
|  | Representativeness | Selection of  non-exposed | Ascertainment  of exposure | Outcome not present at start | Comparability on most important factors | Comparability on other risk factors | Assessment of outcome | Long enough follow-up (median≥1 year) | Adequacy  (completeness) of follow-up |
| Zhai 2021 | ● | ● | ● | ● | ● | ○ | ● | ○ | ● |
| Güç 2022 | ● | ● | ● | ● | ● | ○ | ● | ● | ● |
| Wei 2022 | ● | ● | ● | ● | ● | ● | ● | ● | ● |
| Fang 2023 | ● | ● | ● | ● | ○ | ○ | ○ | ● | ● |
| Mazzella 2023 | ● | ● | ● | ● | ● | ○ | ● | ● | ● |
| Zhao 2023 | ● | ● | ● | ● | ○ | ○ | ● | ● | ● |
| Zhang 2023 | ○ | ● | ● | ● | ● | ○ | ● | ● | ● |
| Cavdar 2024 | ● | ● | ● | ● | ● | ○ | ● | ● | ● |
| Gao 2024 | ● | ● | ● | ● | ● | ● | ● | ● | ● |
| Taylor 2024 | ● | ● | ● | ● | ● | ○ | ● | ● | ● |
